# Identification of genes inducing resistance to ionizing radiation in human rectal cancer cell lines: re-sensitization of radio-resistant rectal cancer cells through down regulating NDRG1

**DOI:** 10.1186/s12885-018-4514-3

**Published:** 2018-05-25

**Authors:** Soon-Chan Kim, Young-Kyoung Shin, Ye-Ah Kim, Sang-Geun Jang, Ja-Lok Ku

**Affiliations:** 1 Laboratory of Cell Biology, Cancer Research Institute, Seoul National University College of Medicine, 103 Daehak-ro, Jongno-gu, Seoul, 03080 Republic of Korea; 2 Department of Biomedical Sciences, Seoul National University College of Medicine, Seoul, 03080 Republic of Korea

**Keywords:** Rectal cancer, Paired rectal cancer cell lines, Establishment, Radiation, Resistance, Gene expression, NDRG1, ERRFI1, Microarray

## Abstract

**Background:**

Resistance to preoperative radiotherapy is a major clinical problem in the treatment for locally advanced rectal cancer. The role of *NDRG1* in resistance to ionizing radiation in rectal cancer has not been fully elucidated. This study aimed to investigate the effect of the reduced intracellular NDRG1 expression on radio-sensitivity of human rectal cancer cells for exploring novel approaches for treatment of rectal cancer.

**Methods:**

Three radio-resistant human rectal cancer cell lines (SNU-61R80Gy, SNU-283R80Gy, and SNU-503R80Gy) were established from human rectal cancer cell lines (SNU-61, SNU-283, and SNU-503) using total 80 Gy of fractionated irradiation. Microarray analysis was performed to identify differently expressed genes in newly established radio-resistant human rectal cancer cells compared to parental rectal cancer cells.

**Results:**

A microarray analysis indicated the RNA expression of five genes (*NDRG1, ERRFI1*, *H19*, *MPZL3*, and *UCA1*) was highly increased in radio-resistant rectal cancer cell lines. Short hairpin RNA-mediated silencing of NDRG1 sensitized rectal cancer cell lines to clinically relevant doses of radiation by causing more DNA double strand breakages to rectal cancer cells when exposed to radiation.

**Conclusions:**

Targeting NDRG1 represents a promising strategy to increase response to radiotherapy in human rectal cancer.

**Electronic supplementary material:**

The online version of this article (10.1186/s12885-018-4514-3) contains supplementary material, which is available to authorized users.

## Background

Colorectal cancer (CRC) is the second most common cause of cancer related deaths in developed countries [1]. Adjuvant chemo- and radio-therapy (RT) with total mesorectal excision (TME) has been a standard approach for rectal cancer patients [2]. Preoperative RT significantly attenuated recurrence rate of rectal cancer especially with a negative circumferential margin [3]. In spite of initial clinical responses, a large portion of patients experience resistance to RT, which is the major cause of rectal cancer-related mortality [4]. Therefore, identifying functionally relevant biomarker to radio-resistant rectal cancer would promote the clinical efficacy of RT.

A wide variety of molecular alterations including chromosomal aberrations and genetic polymorphisms lead to radio-resistance [5]. Although a number of potential targets such as *Survivin* [6] and *TCF4* [7] have been identified, the heterogeneity of radio-resistant rectal cancer emphasizes necessity of various in vitro models representing diverse genetic backgrounds and radio-sensitivity [8]. Thus, we newly established three radio-resistant human rectal cell lines, and analyze their changes in mRNA expression using microarray. Five genes (*NDRG1, ERRFI1*, *H19*, *MPZL3*, and *UCA1*) were selected as potential candidates. Among them, *ERRFI1* and *NDRG1* became final gene of interest as their mRNA and protein expression level was highly increased in radio-resistant rectal cancer cells.

The protein expression of *ERRFI1* (*ERBB* receptor feedback inhibitor, also known as *MIG6* or *GENE33*), which is a negative regulator of *EGFR*, is reported to be down-regulated in skin, breast, pancreatic, ovarian, and liver cancer [9, 10], and augmented protein expression of *EGFR* is known to be involved with radio-resistance [11]. Although *ERRFI1* has been studied with regard to drug-resistance in colorectal cancer [12], its role in radio-resistance has not been studied yet. N-myc downstream-regulated gene 1 (*NDRG1*) has been reported as a possible metastasis suppressor by maintaining the localized E-cadherin and β-catenin in prostate and colon cancer cells [13]. In addition, the neuroblastoma study revealed that overexpression of NDRG1 causes increased level of resistant-related proteins such as MDR, LRP-1, and MRP-1 [14]. Nevertheless, its role in resistance to ionizing radiation in rectal cancer cell lines has not been revealed.

## Methods

### Establishing radio-resistant rectal cancer cell lines and cell culture condition

Seven rectal cancer cell lines (SNU-61, SNU-283, SNU-503, SNU-977, SNU-977R80Gy, SNU-1411, and SNU-1411R80Gy) were provided by the Korean Cell Line Bank (Seoul, Korea). Catalogues numbers of the cell lines were 00061, 00283, 00503, 00977, 00977-RAD, 01411, and 01411-RAD respectively. A total of 80 Gy of fractionated ionizing radiation were irradiated to three rectal cancer cell lines (SNU-61, SNU-283, and SNU-503) over 40 times by using Cesium-137 irradiator. The established radio-resistant rectal cancer cell lines were deposited by Korean Cell Line Bank. The catalogues numbers of the established radio-resistant rectal cell lines are 00061/R80GY, 00283/R80GY, and 00503/R80GY. All cell lines were cultured in RPMI1640 media with 10% FBS and penicillin (100 units/ml)-streptomycin (100μg/ml) (Thermo Fisher Scientific, CA, USA).

### DNA fingerprinting analysis

A DNA fingerprinting analysis is used to authenticate each cell line. The genomic DNA from each cell line was amplified using the AmpFlSTR identifiler PCR amplification kit (Applied Biosystems, Foster City, CA, USA). A single PCR amplified 15 tetranucleotide repeat loci (CSF1PO, D2S1338, D3S1358, D5S818, D7S820, D8S1179, D13S317, D16S539, D18S51, D19S433, D21S11, FGA, TH01, TPOX, and vWA) and Amelogen gender determining marker at loci containing highly polymorphic microsatellite markers. Amplified products were analyzed using an ABI 3730 genetic analyzer (Applied Biosystems). The STR profiles of established radio-resistance rectal cancer cell lines and their parental cell lines are listed in Additional file 1: Table S2.

### Cell counting

3.0 × 10^5^ cells were seeded in 3 ml of culture medium onto 6-well plates. Cells were maintained in humidified incubators at 37 °C in an atmosphere of 5% CO2 and 95% air for 24 h. Cells were exposed to 4 Gy of radiation using Cs-137 irradiator and stained with 0.4% trypan blue. The number of viable cell were counted using the Countess™ cell counting chamber slide (Invitrogen, Carlsbad, CA, USA) and Countess™ automated cell counter (Invitrogen) for 96 h in 24 h intervals. Every process was repeated three times for each cell line.

### Cell viability assay

0.5 × 10^5^ cells were seeded on 96 well plates with 0.5 ml of RPMI1640 media with 10% FBS and 1.1% penicillin for the cell viability assay. Meanwhile, 7.5 × 10^5^ cells were simultaneously seeded on a T75 flask with 15 ml of RPMI1640 media at 10% FBS and 1.1% penicillin for Western Blotting. After 24 h of incubation at 37 °C in a 5% CO2 and 95% air atmosphere, cells were exposed to 12 Gy of Cs-137. In a time course manner (6, 24, 48, and 72 h after irradiation), 10 μl of EZ-Cytox solution (Daeil Lab, Seoul, Korea) was added to each well of 96 well plates. After 2 h of incubation at 37 °C, the optical density was measured at 450 nm by Multiskan™ GO Microplate Spectrophotometer (Thermo Fisher Scientific, Waltham, MA, USA). 1.0 × 10^5^ cells were seeded on 96 well plates with 0.5 ml of RPMI1640 media at 10% FBS and 1.1% penicillin, which were irradiated with 0 and 4 Gy of Cs-137 after 24 h. 50 μl of MTT [3-(4, 5-dimethylthiazol-2-yl)-2, 5-diphenyltetrazolium bromide] (Sigma-Aldrich co., St, Louis, MO, USA) solution diluted with PBS (2.5 mg/ml) was added to each well of the 96-well plates at the same time interval as the cell counting (0 and 96 h). After 4 h of incubation, the MTT solution was removed and 150 μl of DMSO (dimethyl sulfoxide) was added. The absorbance was measured by an ELISA reader (Molecular Devices Co., CA, USA) at 540 nm after being incubated for 15 min at room temperature.

### Cell cycle analysis by FACS

7.5 × 10^5^ cells were seeded on a T75 flask with 15 ml of RPMI1640 media with 10% FBS and 1.1% penicillin. After 24 h of incubation at 37 °C in an atmosphere of 5% CO2 and 95% air, cells were exposed to 4 Gy of Cs-137 and collected 96 h after irradiation. Collected cells were fixed with 70% ethanol and incubated at 4 °C for 24 h. After washing with cold DPBS, cells were stained with propidium iodide (PI) (100 μg/ml) (Sigma-Aldrich Co.) and RNase A (10 mg/ml) (Intron biotechnology, Gyeonggi, Korea) for 30 min on ice. Then, a fluorescence-activated cell sorter (FACS CantoII™, BD, NJ, USA) was used to analysis the cell cycle phases.

### Colony forming assay

1.5 ml of 0.5% noble agar (BD Difco™, Franklin Lakes, NJ, USA) in RPMI1640 with 10% FBS was solidified at the bottom of each individual well in a 6-well plate. 3.5 × 10^3^ cells in 500 μl of RPMI1640 media with 10% FBS and 1.1% penicillin were mixed with 500 μl of 0.4% agar and spread on the bottom agar. After 24 h of incubation at 37 °C in an atmosphere of 5% CO2 and 95% air, the plate was irradiated with 4 Gy of Cs-137. Every 5 d 300 μl of RPMI1640 media at 10% FBS and 1.1% penicillin was added to prevent desiccation of agar media. Three weeks later, the colonies were stained with 500 μl of 0.05% crystal violet and counted under a phase-contrast microscope. The number of colonies from triplicated wells was averaged.

### Microarray analysis

Total RNA was extracted from induced radio-resistant and parental cell lines using TRIzol (Invitrogen, Carlsbad, CA, USA) and purified with the RNeasy Mini Kit (Qiagen, Hilden, Germany) according to manufacturer’s instructions. The RNA integrity was assessed by an Agilent 2100 Bioanalyzer (Agilent, Palo Alto, CA, USA). High quality RNA (RNA integrity number > 9.0) was used for the gene expression microarray analysis in which 100 ng of total RNA was processed for biotin labeled target preparation and hybridization to the Affymetrix Gene 1.0 ST array according to manufacturer’s instructions in order to perform the gene expression profiling experiments (Affymetrix, Inc., Santa Clara, CA, USA). After 16 h of hybridization at 45 °C and rotating at 60 rpm, the arrays were washed and stained on a GeneChip Fluidics Station (Affymetrix, Inc.) and scanned using the Gene Chip Scanner 3000 (Affymetrix, Inc.). The CEL intensity data extracted by GCOS (Gene Chip Operating Software) was used for the data analysis and raw data were processed using the Affymetrix® Expression Console software with the default RMA parameters.

### RNA isolation and cDNA synthesis

Cells were collected by trypsinization and suspended in TRIzol (Invitrogen, Carlsbad, CA, USA). The total RNA was isolated with the RNeasy Mini Kit (Qiagen, Hilden, Germany) according to manufacturer’s instructions. For cDNA synthesis, QuantiTect Reverse Transcription Kit (Qiagen) is used. 1 μg of total RNA, 2 μl of gDNA Wipeout Buffer, and DEPC water filled up to 14 μl were mixed together and incubated at 42 °C for 2 min. The mixture was then blended with Quantiscript RT Buffer, RT Primer Mix, and Quantiscript® Reverse Transcriptase, and incubated at 42 °C for 45 min. The mixture was further incubated at 95 °C for 2 min and cooled down to room temperature.

### Reverse transcriptase-PCR (RT-PCR)

Synthesized cDNA was diluted to 100 ng/μl using distilled water. 1 μl of 100 ng/μl cDNA was amplified in 14 μl of a PCR mixture that contained 1.5 μl of 10 x PCR buffer (with MgCl_2_), 0.5 μl of dNTP, 0.25 μl of forward primer (10 pmol/ul), 0.25 μl reverse primer (10 pmol/ul), 11.42 μl of distilled water, and 0.08 μl of i-Taq DNA polymerase (500 units) (Intron biotechnology, Gyeonggi, Korea). The primer sequences that were used in this study are listed in Additional file 1: Table S1. RT-PCR was performed using a programmable thermal cycler (PCR System 9700, Applied Biosystems; Foster City, CA, USA) and the RT-PCR products were fractionated on a 1.5% agarose gel containing ethidium bromide (EtBr).

### Real-time PCR

Synthesized cDNA was diluted to 10 ng/μl and each primer concentration was optimized. 1 μl of cDNA was mixed with 5.0 μl of Master Mix (Applied Biosystems), distilled water, and optimized volume of each forward and reverse primer. The primer sequences were the same as the ones used in RT-PCR, as listed in Additional file 1: Table S1. A real-time PCR analysis was performed with the 7900HT Fast Real-Time PCR System (Life Technologies Co., Carlsbad, CA, USA) and the results were normalized to the housekeeping gene, β-actin, and the cycle threshold (Ct) values were extracted.

### Protein isolation and western blotting

0.5 × 10^5^ cells were seeded on 96 well plates with 0.5 ml of RPMI1640 media with 10% FBS and 1.1% penicillin for the cell viability assay while 7.5 × 10^5^ cells were simultaneously seeded on a T75 flask with 15 ml of RPMI1640 media with 10% FBS and 1.1% penicillin for Western Blotting. Cells were harvested with a cell scraper after washing with cold PBS. Whole protein was extracted with EzRIPA buffer (ATTO Co., Tokyo, JAPAN) supplied with 1% protease inhibitor and 1% phosphatase inhibitor in accordance with the cell viability assay time frame. The volume of lysis buffer was adjusted to the number of cells collected in each vial. The protein concentration was determined by SMART™ micro BCA protein assay kit (Intron biotechnology, Gyeonggi, Korea). Equal amounts of protein were loaded on 4–15% Mini-PROTEAN TGX™ Precast Gels (BIO-RAD, Hercules, CA, USA) and blotted at 50 voles for 2 h. Proteins were then transferred to Trans-Blot Turbo™ Transfer Pack (BIO-RAD) using Trans-Blot Turbo™ Transfer System V1.02 machine (BIO-RAD) at 2.5 Amp and 25 Volt. The membrane was incubated in 2.5% skim milk containing 0.5% Tween 20 for an hour at room temperature. Primary antibodies against NDRG1 (abcam, Cambridge, United Kingdom) (1:5000), ERRFI1 (Santa Cruz Biotechnology, Inc., Santa Cruz, CA, USA) (1:1000), PARP (BD Biosciences, San Jose, CA, USA) (1:1000), Caspase-3 (abcam, Cambridge, United Kingdom) (1:2000), and β-actin (Applied Biological Materials Inc., Richmond, BC, Canada) (1:5000) were diluted with 1.5% skim milk (BD Biosciences, CA, USA) containing 0.5% Tween 20 and introduced to the membrane. After 2 h at room temperature, Peroxidase conjugated mouse or rabbit IgG antibody (Jackson Immunoresearch, West Grove, PA, USA) (1:5000) was added as a secondary antibody. A chemiluminescent working solution, WESTZOL™ (Intron biotechnology), was decanted into the membrane, which was then exposed to Fuji RX film (Fujifilm, Tokyo, Japan) for 1–5 min.

### Immunofluorescent staining

Cells were seeded on chambered coverglass (Thermo Fisher Scientific, MA, USA) at densities of 3 × 10^3^ to 2 × 10^4^ cells/mL in culture media, according to diverse cell growth rates and desirable cell confluency. After 72 h, cells were washed with DPBS three times before being fixed and permeabilized using BD Perm/Wash™ (BD bioscience, CA, USA). After cells were washed with washing solution (BD bioscience, CA, USA), a PBS solution containing 2% FBS (GE Healthcare Life Sciences, Buckinghamshire, UK) was added. Primary antibodies that were used for Western Blotting were applied for immunofluorescent staining at dilution factors of NDRG1 (1:50), ERRFI1 (1:100), PARP (1:100), and Caspase-3 (1:100). 1 to 2 h after the primary antibody was applied, cells were washed with cold PBS containing 0.05% of tween 20. A conjugated secondary antibody (Thermo Fisher Scientific, MA, USA) was applied for 1 to 2 h in accordance with matched species. After cells were washed with cold PBS containing 0.05% of tween 20, distilled water with 1× DAPI and Rhodamine-conjugated Phalloidin (Sigma-Aldrich, MO, USA) was added for 20 min. Finally, the cells were washed with DPBS three times and viewed using a LSM800 Confocal Laser Scanning Microscope (Carl Zeiss, Oberkochen, Germany).

### Knockdown of ERRFI1 expression by siRNA transfection

2.0 × 10^5^ cells of SNU-503R80Gy with 1 ml of culture media were seeded in a 6-well plate and transfected with control siRNA and *ERRFI1* siRNA (No#:1048480) (Bioneer, Alameda, CA, USA) with lipofectamine 2000 (Invitrogen, Carlsbad, CA, USA) at a final concentration of 40 nM siCONT and 40 nM si*ERRFI1* in Opti-MEM medium for 6 h. The media was then replaced with an equal volume of RPMI1640 (Gibco) without antibiotics. Consequently, the cells were collected for cell counting and cell viability assay for confirmation of the mRNA level.

### Knockdown of NDRG1 expression by shRNA transduction

4 × 10^4^ of 293FT cells with 10 ml of DMEM media supplied with 10% of FBS and 1% of penicillin streptomycin were seeded on 100 pi tissue culture. After 24 h of incubation, the culture media was changed to 10 ml of Opti-MEM, and ViraSafe™ Lentiviral Packaging System, Pantropic (CELL BIOLABS, INC., San Diego, CA, USA) with short hairpin RNA targeting *NDRG1* (Sigma-Aldrich co., St, Louis, MO, USA) was treated with Lipofectamine 3000 (Invitrogen) in accordance with manufacturer’s protocol. After 48 h, the viral soup was harvested and filtered through a 0.45 μm pored filter (Sartorius Stedim Biotech SA, Göttingen, Germany). The resulting harvested viral soup was aliquoted into a 1.5 mL tube and kept at − 70 °C. 1 × 10^5^ cells/ml of SNU-503R80Gy cells were seeded on 24 well tissue culture plates in 0.5 ml of RPMI1460 medium and incubated at 37 °C overnight. Viral transduction was performed using ViraDuctim™ (CELL BIOLABS, INC., San Diego, CA, USA) according to manufacturer’s protocol. The efficacy of shRNA on down-regulating NDRG1 was confirmed by Western Blot.

### Statistical analysis

All acquired data in this study were analyzed by GraphPad Prism 5.0 and expressed as mean ± standard deviation. A comparison between the two cell lines (SNU-503 and SNU-503R80GY) was performed by a two-way variance analysis (two-way ANOVA) with the radiation time and dose as dependent variables.

## Results

### Phenotypical changes of the radio-resistant rectal cancer cell line (SNU-503R80Gy) compared to its parental cell line (SNU-503)

**Fig. 1:**
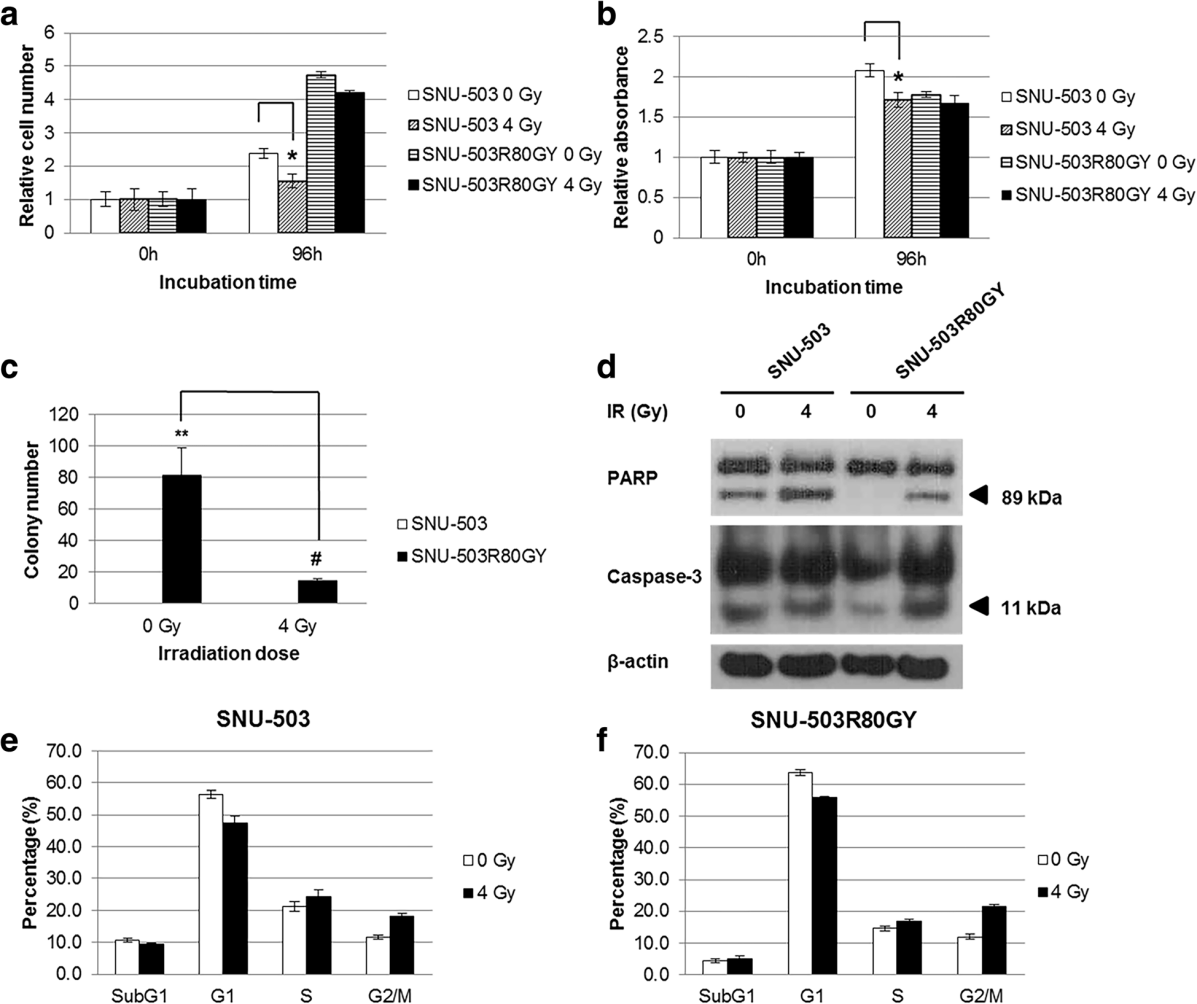
Cellular changes in the induced radio-resistant rectal cancer cell lines based on the cell proliferation assay, colony forming assay, apoptotic assay and cell cycle analysis. Cell counting **a** and MTT assay **b** were performed in the SNU-503 and SNU-503R80Gy cell lines 96 h after irrdiation with 0 and 4 Gy. **c** For the colony forming assay, the number of colonies were averaged from three wells. **d** Cellular apoptosis was observed by western blot analysis with cleaved PARP and Caspase-3 protein levels. Cell cycle analysis was performed by FACS (**e** and **f)**. The entire experiments were repeated three times. (* *P* < 0.05: comparison of cell proliferation between when the cell line was irradiated with 0 and 4 Gy, ** *P* < 0.0001: comparison of the number of colonies between the parental and the induced radio-resistant rectal cancer cell lines, # *P* < 0.0002: comparison of the number of colonies between when the cell line was irradiated with 0 and 4 Gy of irradiation)

Among the three pairs of radio-resistant rectal cancer cell lines, the SNU-503 and SNU-503R80Gy cell lines were selected for the functional study. Under normal growth condition, radio-resistant rectal cancer cells (SNU-503R80Gy) grew 1.6 times faster than its parental cells (SNU-503). When exposed to 4 Gy, the growth rate of radio-resistant rectal cancer cells was decreased by 11.0% whereas that of parental cell line was declined by 35.1% (Fig. 1a) (* *P* < 0.05). The higher survival rate of radio-resistant rectal cancer cells under irradiation was confirmed again by MTT assay (Fig. 1b) (* P < 0.05). A soft agar colony formation assay was conducted. Under normal growth condition, SNU-503R80Gy formed 82 visible colonies in average while SNU-503 formed no colonies. Under 4 Gy, SNU-503R80Gy formed 16 visible colonies in average whereas SNU-503 formed no colonies (Fig. 1c). The cleavage of poly-(ADP-ribose) polymerase (PARP) and caspase-3 was also examined under 4 Gy irradiation. The basal level of cleaved PARP and caspase-3 was declined in radio-resistant rectal cancer cells compared to that of rectal cancer cells. Although cleavage level of both PARP and caspase-3 was increased after 4 Gy irradiation, there was no evident difference between radio-resistant and its parental rectal cancer cells (Fig. 1d). A cell cycle analysis was performed to check if DNA damage induced cellular senescence. Overall, the G2/M phase of both SNU-503 and SNU-503R80Gy cell lines increased by 29.3 and 23.7%, respectively. However, there was no significant difference between the two cell lines (Fig. 1e and f).

### Differential mRNA expression in the induced radio-resistant rectal cancer cell lines

**Fig. 2:**
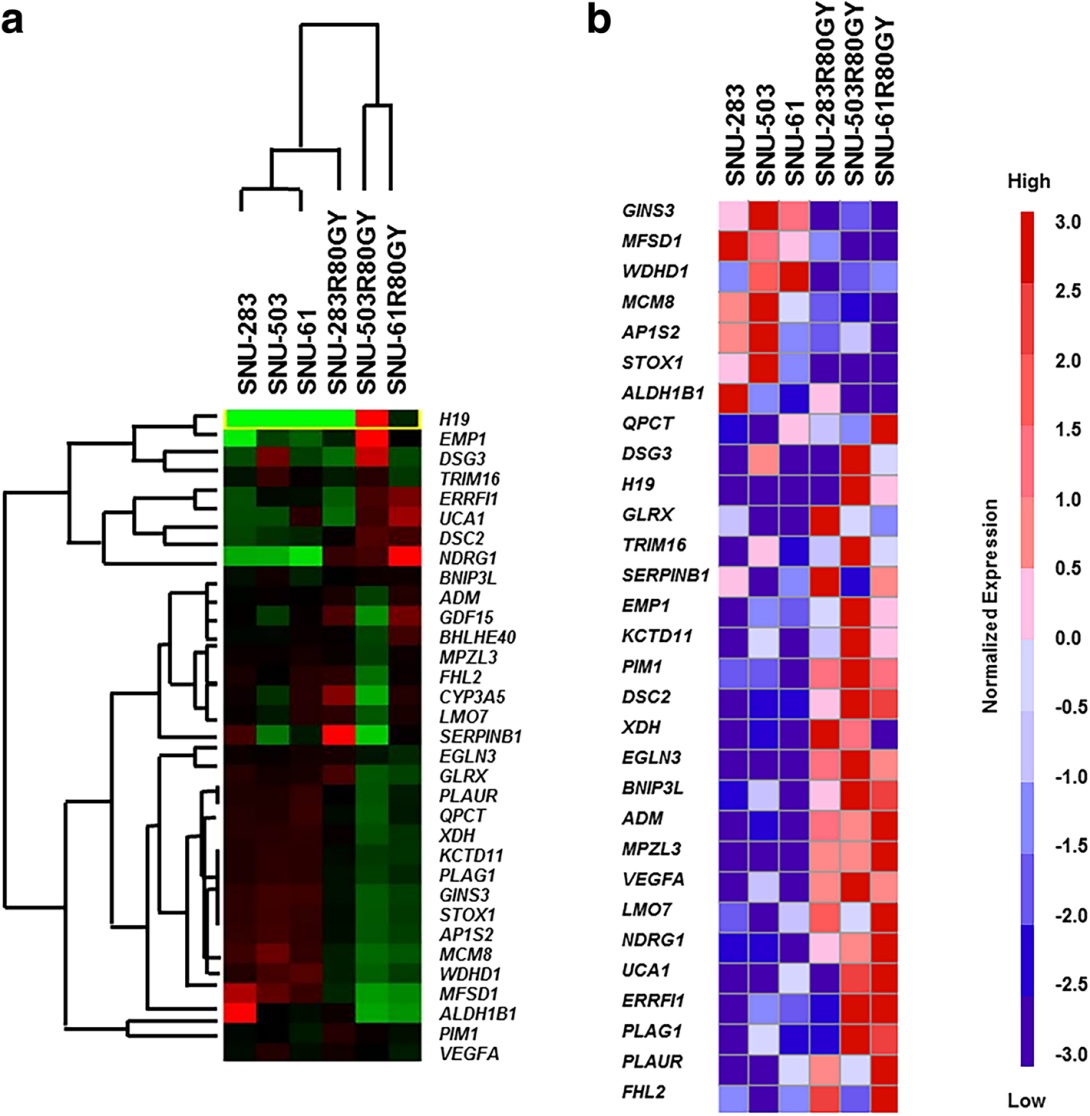
Microarray analysis of the parental and induced radio-resistant rectal cancer cell lines. **a** Thirty-three genes with fold change (FC) > 2 as the threshold of differential expression were identified and clustered according to their mRNA expression level. **b** After normalization, 26 genes had more than one-fold increase, and seven genes had more than one-fold decrease

A microarray was performed on three pairs of parental and induced radio-resistant rectal cancer cell lines (SNU-61, SNU-61R80Gy, SNU-283, SNU-283R80Gy, SNU-503, and SNU-503R80Gy) in order to identify differential mRNA expressions. When classified with fold change (FC) > 2 as the threshold of differential expression in the induced radio-resistance rectal cancer cell lines, 26 genes had more than one-fold increase, and seven genes had more than one-fold decrease (Fig. 2). Clustering was done to categorize genes and cell lines according to their FC. All three parental cell lines (SNU-61, SNU-283, and SNU-503) were clustered together. Among the induced radio-resistant rectal cancer cell lines, SNU-283R80Gy had analogous mRNA expression pattern with the parental cell lines. SNU-61R80Gy and SNU-503R80Gy were clustered separately (Fig. 2a). Most of the differently expressed genes were related to hypoxia, oxygen levels, oxidation reduction, regulation of epithelial cell differentiation, and cell-cell adhesion according to the gene ontology analysis (Table 1).

**Table 1:** Gene ontology analysis of altered genes in radio-resistant rectal cancer cell lines

Probe Set ID	Gene symbol	GO_BP			SNU-61		FC	SNU-283		FC	SNU-503		FC
					P	R		P	R		P	R	
7912157	*ERRFI1*				288	1288	4.5	52	206	4.0	334	1215	3.6
7938390	*ADM*	●	◎		178	746	4.2	185	524	2.8	222	469	2.1
7945680	*H19*				79	1562	19.8	65	265	4.1	74	6924	93.8
7952036	*MPZL3*				100	509	5.1	86	297	3.5	75	324	4.3
7954090	*EMP1*				654	1493	2.3	66	1171	17.8	811	3459	4.3
7978544	*EGLN3*	●	◎	◑	114	304	2.7	117	376	3.2	93	515	5.6
8004360	*KCTD11*				44	95	2.1	37	78	2.1	82	196	2.4
8020762	*DSG3*	◯			53	450	8.5	29	98	3.4	800	1741	2.2
8022711	*DSC2*	◯			589	1463	2.5	481	1017	2.1	561	1651	2.9
8026490	*UCA1*				485	1434	3.0	38	121	3.1	37	1153	31.3
8027002	*GDF15*				318	1203	3.8	332	843	2.5	73	255	3.5
8037374	*PLAUR*				109	228	2.1	59	152	2.6	49	115	2.4
8041508	*QPCT*				90	197	2.2	26	58	2.2	10	48	4.8
8051322	*XDH*	◑	◐		40	101	2.5	44	142	3.2	51	105	2.1
8077441	*BHLHE40*				240	904	3.8	193	470	2.4	178	476	2.7
8113214	*GLRX*	◑			32	90	2.8	116	459	4.0	31	133	4.3
8119161	*PIM1*				128	608	4.7	238	637	2.7	265	851	3.2
8123598	*SERPINB1*				443	1015	2.3	856	1918	2.2	81	271	3.3
8141328	*CYP3A5*	◑			350	785	2.2	283	998	3.5	16	46	3.0
8145454	*BNIP3L*				158	811	5.1	269	551	2.0	403	944	2.3
8150881	*PLAG1*				29	136	4.6	18	37	2.0	76	165	2.2
8153002	*NDRG1*				208	3499	16.8	463	1615	3.5	436	2010	4.6
8012953	*TRIM16*	◐			159	337	2.1	86	263	3.0	384	866	2.3
8054377	*FHL2*				165	505	3.1	157	451	2.9	49	124	2.5
8119898	*VEGFA*	●	◎		168	550	3.3	210	538	2.6	396	794	2.0
7969438	*LMO7*	●			273	666	2.4	179	557	3.1	48	320	6.6
7927915	*STOX1*				29	14	−2.1	47	17	−2.8	108	18	−5.9
7979281	*WDHD1*				288	114	−2.5	109	54	−2.0	238	96	−2.5
7996211	*GINS3*				115	41	−2.8	88	38	−2.3	159	58	−2.8
8017210	*AP1S2*				43	15	−2.9	82	32	−2.6	134	45	−3.0
8060813	*MCM8*				259	122	−2.1	340	170	−2.0	526	165	−3.2
8083656	*MFSD1*				665	301	−2.2	1212	530	−2.3	865	354	−2.4
8155327	*ALDH1B1*	◑			214	81	−2.6	1622	566	−2.9	299	133	−2.3

Genes that differently expressed more than 2-folds in radio-resistant rectal cancer cell lines were analyzed with gene ontology. (P: Parental rectal cancer cell line, R: Radio-resistant rectal cancer cell line, FC: Fold change, ●: Response to hypoxia, ◎: Response to oxygen levels, ◑: Oxidation reduction, ◐: Regulation of epithelial cell differentiation, and ◯: Cell-cell adhesion)

**Fig. 3:**
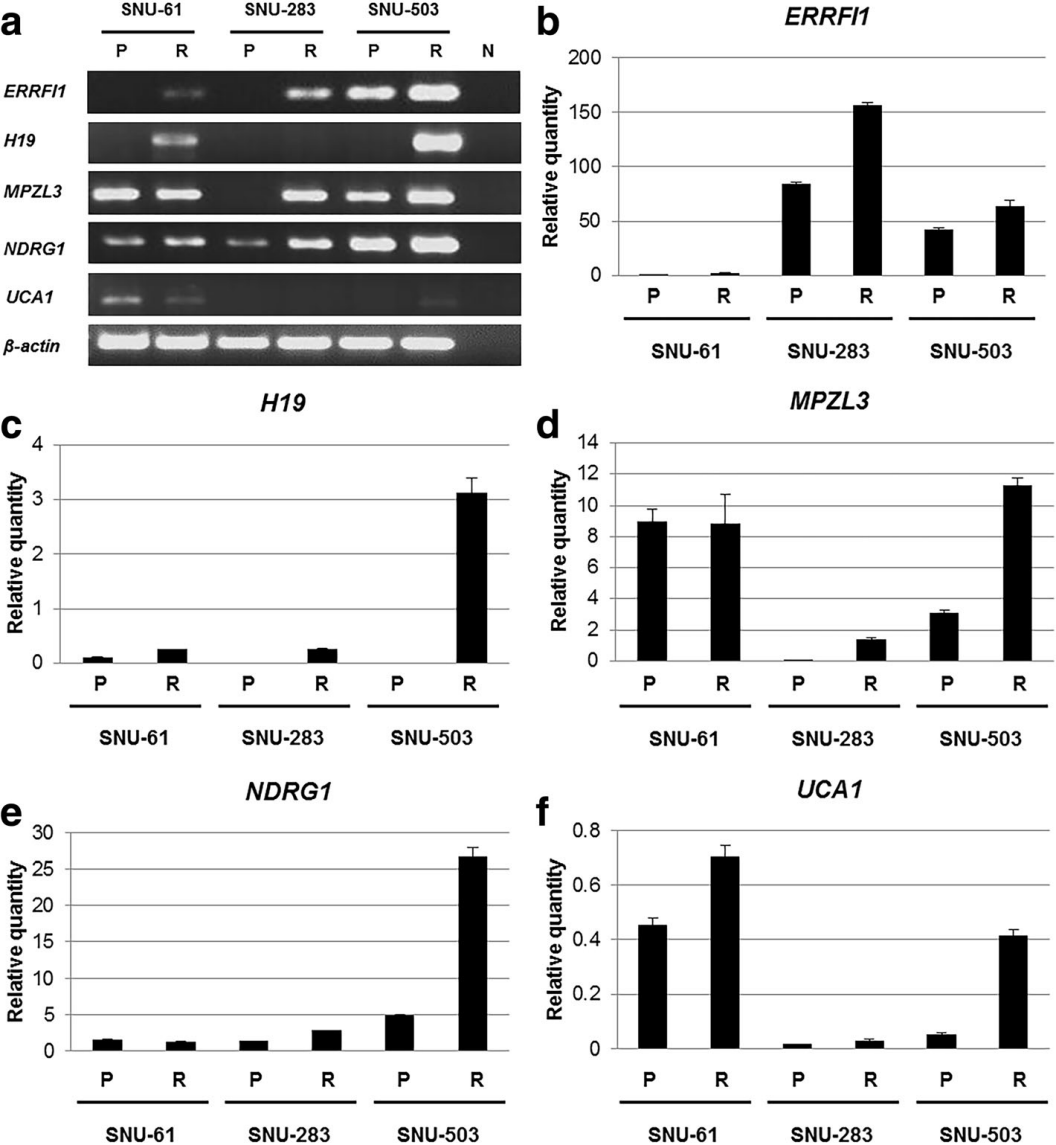
Potential target genes for acquiring radio-resistance were further sorted with FC > 3 threshold, and five genes (NDRG1, ERRFI1, H19, MPZL3, and UCA1) were selected. RT-PCR (**a**) and real-time PCR (**b**, **c**, **d**, **e**, and **f**) confirmed that the mRNA expression patterns of the five genes were analogous to the microarray analysis (P: parental rectal cancer cell line, R: radio-resistant rectal cancer cell line, N: negative control)

Potential target genes for acquiring radio-resistance were further sorted with FC > 3 threshold, and five genes (*NDRG1*, *ERRFI1*, *H19*, *MPZL3*, and *UCA1*) were selected (Table 2). Real-time PCR (Fig. 3b, c, d, e, and f) confirmed that the mRNA expression patterns of the five genes were analogous to the microarray analysis, and the mRNA expression level of *NDRG1* and *ERRFI1* was specifically increased in the induced radio-resistant rectal cancer cells.

**Table 2:** List of genes that were up-regulated more than 3-folds in radio-resistant rectal cancer cell lines

Gene symbol	Description	FC^a^
*ERRFI1*	ERBB receptor feedback inhibitor 1	3.6
*H19*	Imprinted maternally expressed transcript	4.1
*MPZL3*	Myelin protein zero-like 3	3.5
*NDRG1*	N-myc downstream regulated 1	3.0
*UCA1*	Urothelial cancer associated 1	3.5

Five candidate genes were selected for functional study in accordance with 3-folds up-regulation in the induced radio-resistant rectal cancer cell lines

*FC* Fold change

^a^the lowest fold change out of three paired rectal cancer cell lines

### ERRFI1 as the radio-resistant candidate marker gene

**Fig. 4:**
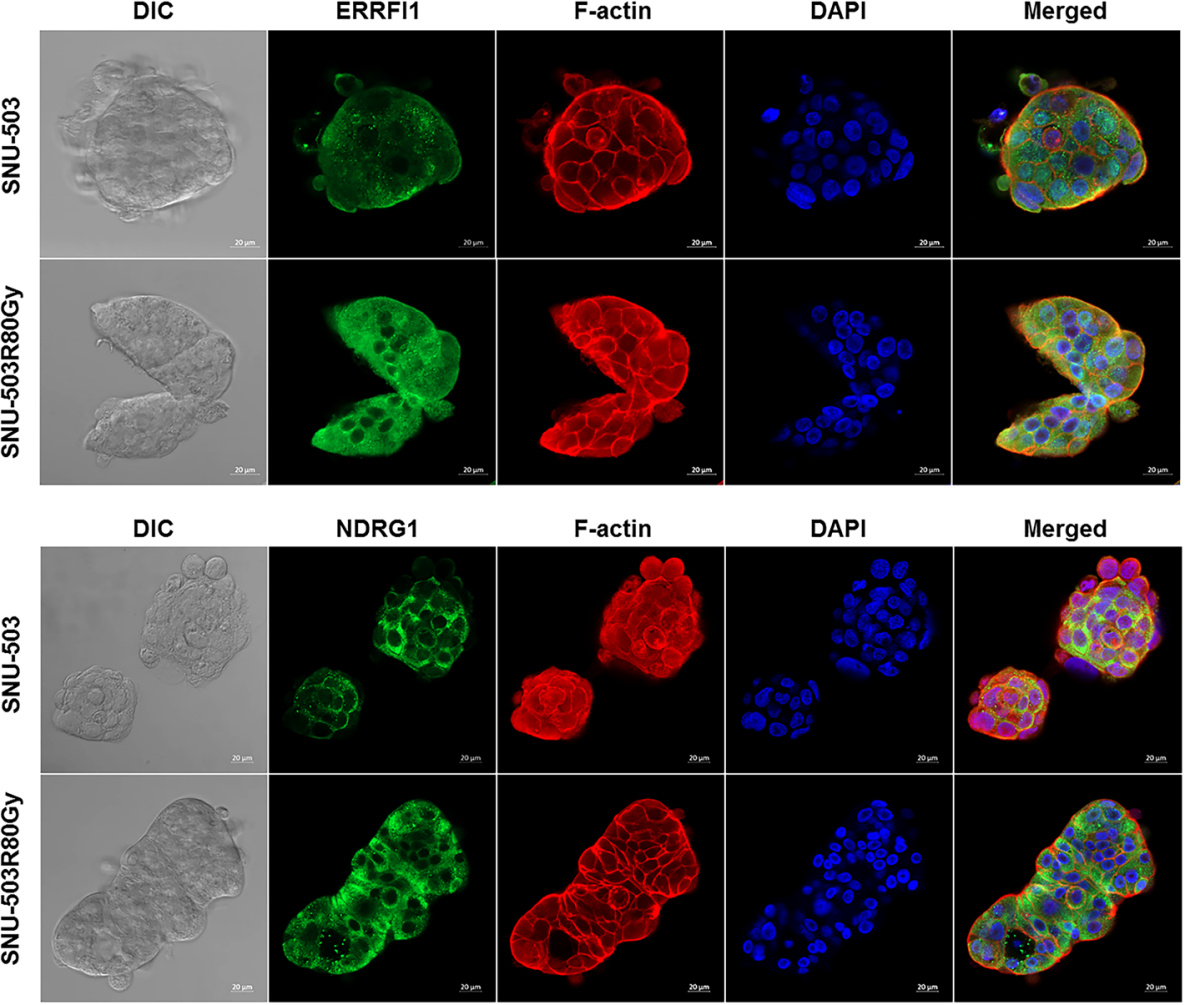
Subcellular localization and expression of the ERRFI1 and NDRG1 proteins in SNU-503 and SNU-503R80Gy

**Fig. 5:**
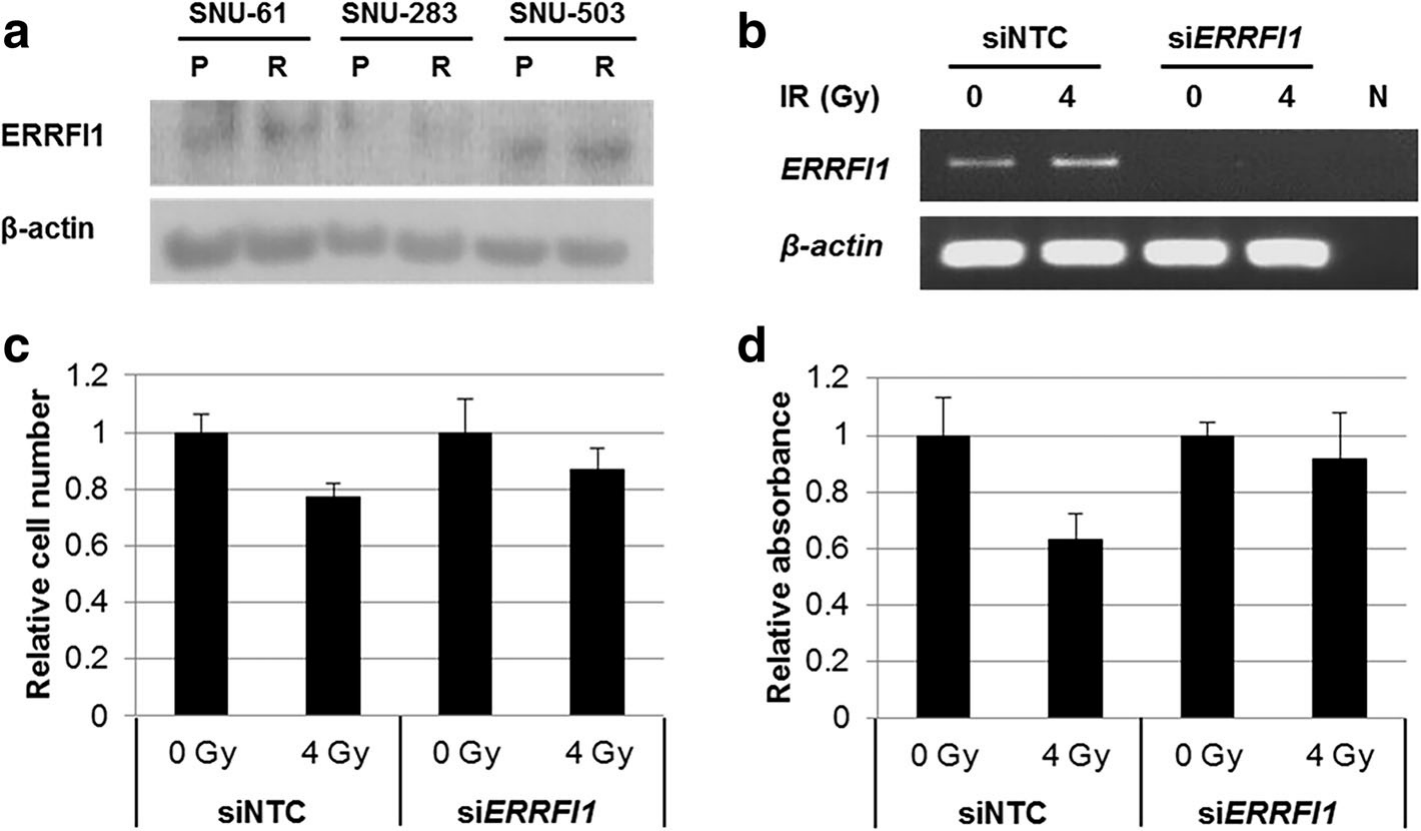
Expression level of the ERRFI1 and its role in cell proliferation under irradiation in the SNU-503 and SNU-503R80GY cell lines. **a** The protein expression of ERRFI1 in three pairs of the induced rectal cancer cell lines was confirmed by Western Blot analysis (P: Parental rectal cancer cell line, R: Radio-resistant rectal cancer cell line) were performed. **b** The knock-down efficiency of siERRFI1 in SNU-503R80Gy cell line was accessed by RT-PCR. Cell counting **c** and MTT assay **d** were performed 96 h after siERRFI1 transfection (N: Negative control)

The protein expression of *ERRFI1* in three pairs of the induced rectal cancer cell lines was confirmed by Western Blot analysis (Fig. 5a). Although the increased protein level in SNU-283R80Gy compared to its parental cell lines was observed, it was obscure to determine the protein expression in the SNU-61 and SNU-503 pairs. In order to further verify the protein expression and cellular localization of ERRFI1, immunofluorescent staining was performed in SNU-503 and SNU-503R80Gy cell lines (Fig. 4). ERRFI1 was mainly localized in cytoplasmic regions and clearly augmented in SNU-503R80Gy. To confirm the function of overexpressed *ERRFI1*, si*ERRFI1* was transfected into the SNU-503R80Gy cell line. The knock-down efficiency of si*ERRFI1* was accessed by RT-PCR (Fig. 5b). When *ERRFI1* was down-regulated, there was no significant change to cell proliferation based on cell counting and MTT assay after 0 and 4 Gy of irradiation (Fig. 5c and d).

### NDRG1 as the radio-resistant candidate marker gene

**Fig. 6:**
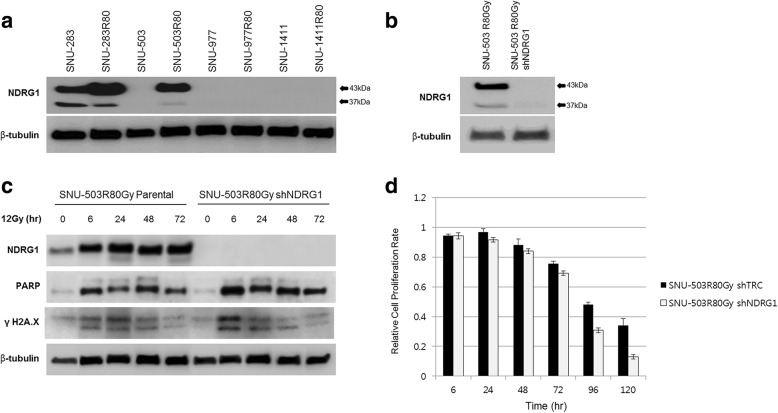
The effect of down-regulated NDRG1 in the radio-resistant rectal cancer cells. **a** Western blot analysis was performed to confirm the basal protein expression of NDRG1. SNU-283R80Gy and SNU-503R80Gy cells had increased level of NDRG1 compared to their parental cells. **b** Knockdown efficacy of NDRG1 with short-hairpin RNA was confirmed with Western blot analysis. **c** Down-regulated NDRG1 increased the sensitivity of radio-resistance rectal cancer cells to irradiation. The amount of cleaved PARP and phosphorylated gamma H2AX was augmented when the expression of NDRG1 was decreased. **d** Cell Proliferation rate was decreased when NDRG1 was down-regulated

**Fig. 7:**
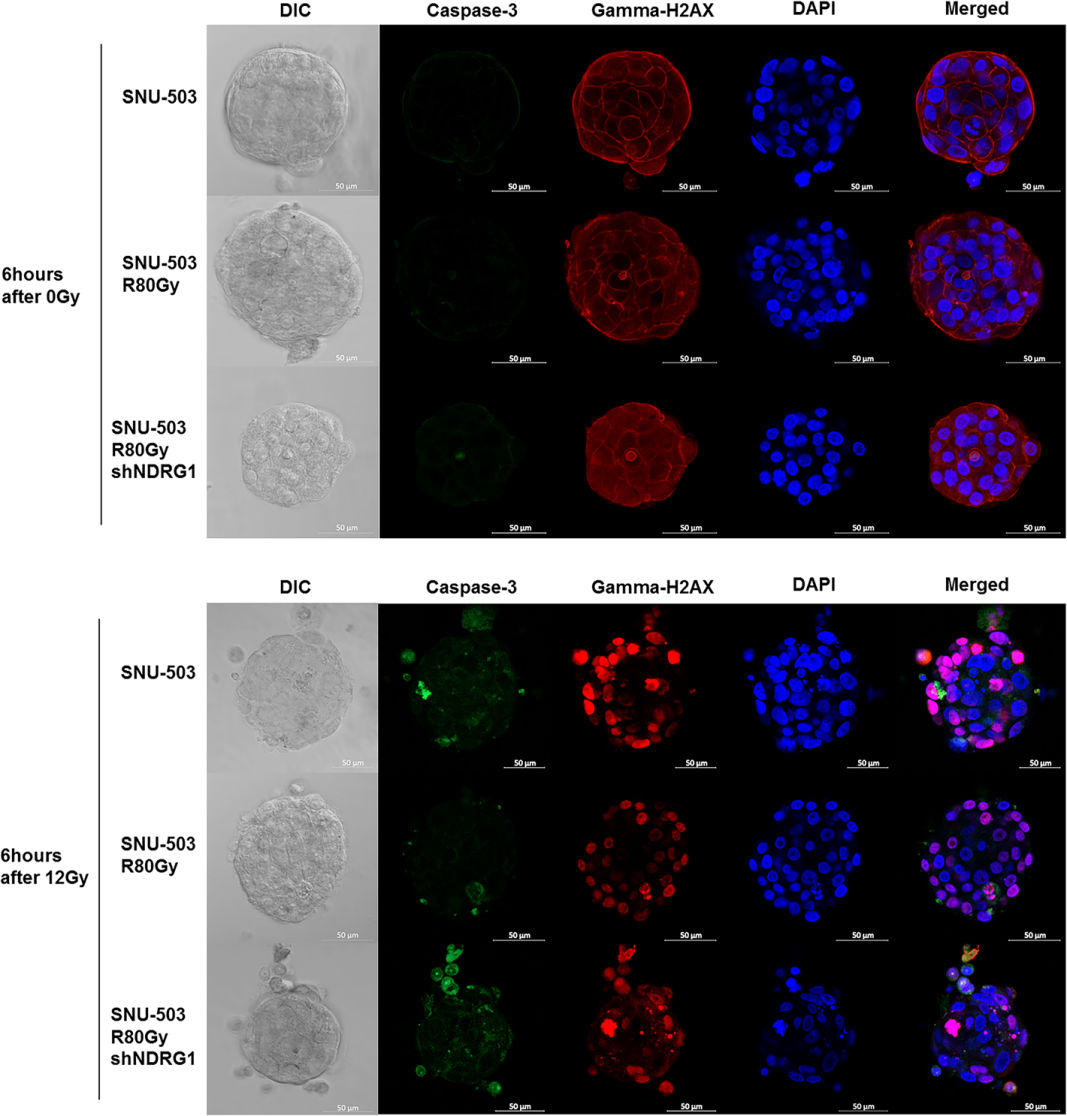
Six hours after 12 Gy irradiation, immunocytochemistry revealed that both active forms of caspase-3 and phosphorylated gamma H2AX were expressed in SNU-503, SNU-503R80Gy and SNU-503R80Gy shNDRG1

**Fig. 8:**
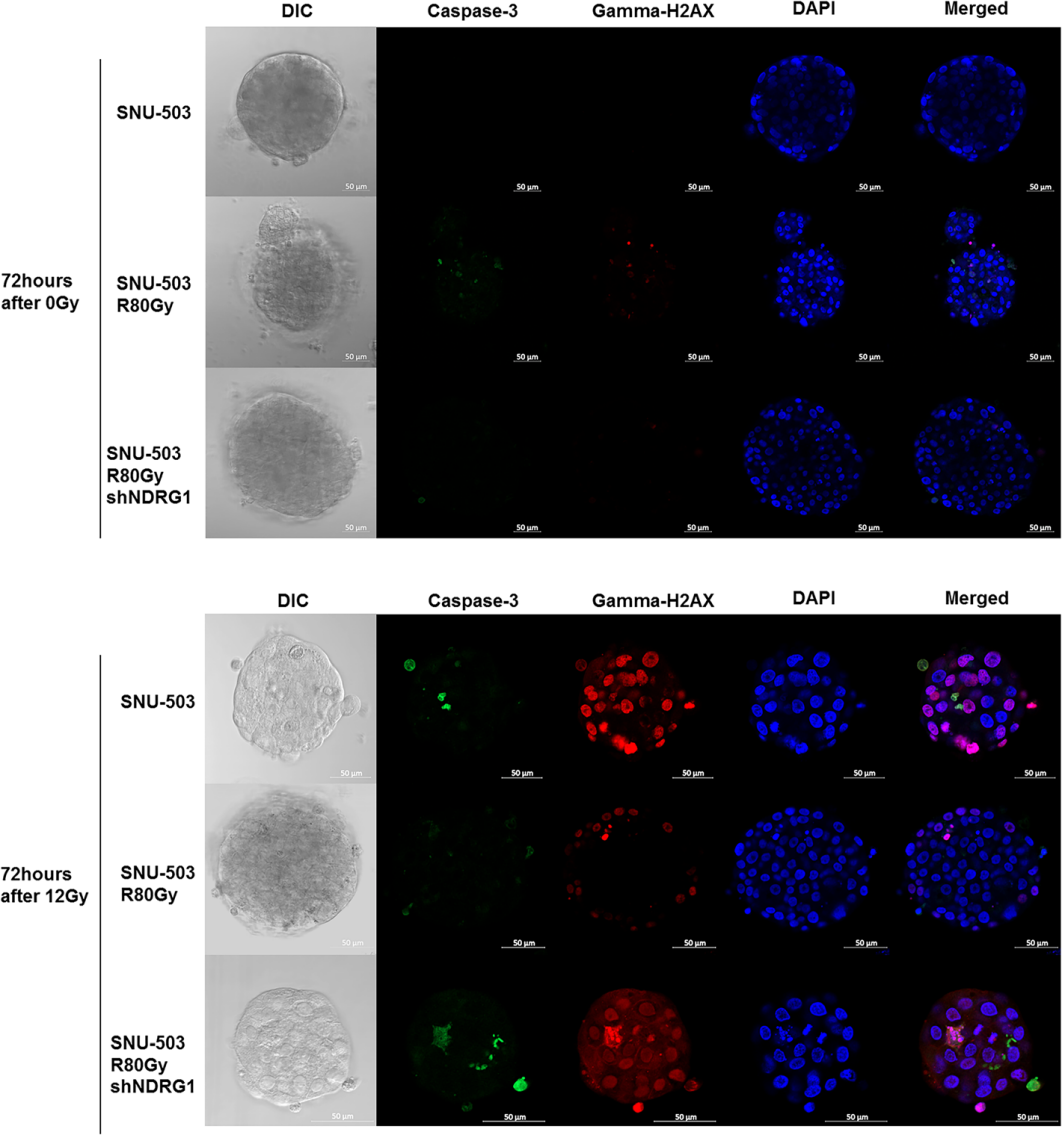
Seventy-two hours after 12 Gy irradiation, immunocytochemistry revealed both active forms of caspase-3 and phosphorylated gamma H2AX were decreased in SNU-503R80Gy cells whereas SNU-503 and SNU-503R80Gy NDRG1 cells strongly expressed both active forms of caspase-3 and phosphorylated gamma H2AX

The SNU-61 and SNU-61R80Gy cell lines were excluded from the functional study due to their slow growth rates. Instead, previously established radio-resistant rectal cell lines (SNU-977, SNU-977R80Gy, SNU-1411, and SNU-1411R80Gy) were included to screen the basal protein level of NDRG1 in the radio-resistance cell lines compared to their parental cell lines. Only SNU-283R80Gy and SNU-503R80Gy showed increased protein levels for NDRG1 compared to their parental cells. Due to the intrinsic NDRG1 expression of the SNU-283 cell line, SNU-503 and SNU-503R80Gy were selected for the further functional study (Fig. 6a). The protein expression of NDRG1 was suppressed with short hairpin RNA targeting *NDRG1*, and the knock-down efficacy was accessed by Western Blot analysis (Fig. 6b). Cells were exposed to 12Gy of radiation and harvested at various time intervals (6, 24, 48, and 72 h). Immunocytochemistry revealed that both active forms of caspase-3 and phosphorylated gamma H2AX were decreased in SNU-503R80Gy cells compared to SNU-503 cells. When NDRG1 was down-regulated in SNU-503R80Gy, cells were damaged again from ionizing radiation and both active forms of caspase-3 and phosphorylated gamma H2AX were detected in a similar level with SNU-503, naïve cells (Figs. 7, and 8). Western Blot analysis revalidated that SNU-503R80Gy with down-regulated NDRG1 was more sensitive to initial damage induction (6–24 h after irradiation) than SNU-503R80Gy parental cells (Fig. 6c and 7). After 72 h, SNU-503R80Gy parental cells repaired damage more effectively than SNU-503R80Gy shNDRG1 cells (Fig. 6c and 8). This was re-confirmed by time-dependent cell proliferation assay. Until 72 h after irradiation, the proliferation rate of SNU-503R80Gy cells with mock vector and shNDRG1 was similar. In 96 h, the proliferation rate of SNU-503R80Gy shNDRG1 was declined significantly (Fig. 6d).

## Discussion

Preoperative radiotherapy combined with total mesorectal excision for resectable rectal cancer has been performed as a prevalent therapeutic and preventative measure for treating colorectal cancer [2]. Preoperative short-term radiotherapy reduced 10-year local recurrence by more than 50% from just surgery alone and significantly improved the 10-year survival in patients with a negative circumferential margin [3]. Nevertheless, patients who experience tolerance to radio-therapy still suffer from relapsed rectal cancer [4]. While numerous genes have been screened for explaining resistance to radiotherapy, the heterogenetic background of recurred rectal cancer after preoperative irradiation accentuates the need for more databases that represent various genetic landscapes.

In this study, three human rectal cancer cell lines (SNU-61, SNU-283 and SNU503) were exposed to repeated 4 Gy dose fractions and allowed to recover to a set confluence of 70% in between fractions. Increased growth rate and colony forming ability are known as general consequence of fractionated radiation exposure and are often involved in a modification of cell cycle distribution [15]. The cumulative exposure of human rectal cancer cells to 80 Gy fractionated radiation resulted in the generation of a sub-line with a significantly increased proliferation rate and clonogenic survival potential following radiation exposure, when compared to mock irradiated, aged-matched cells (Fig. 1a, b and c).

It is reported that radio-resistance is maintained by G2/M cell cycle arrest [16]. Compared to the SNU-503, G2/M phase was increased more in SNU-503R80Gy when exposed to 4 Gy-dose radiation. This may suggest that SNU-503R80Gy cells resisted the damage from ionizing irradiation by arresting its cell cycle at the G2/M phase. Further evaluation of the underlying mechanisms for the amplification of the G2/M-phase cell population is warranted.

Cleaved PARP and active form of the caspase-3 has become a useful hallmark of apoptosis [17]. Compared to the SNU-503, the basal protein expression of both cleaved PARP and the active form of the caspase-3 was decreased in SNU-503R80Gy (Fig. 1d). Given that both SNU-503 and SNU-503R80Gy grew as irregular convex aggregates in which cells at the center of the aggregates may experience hypoxic damage, a decreased cleaved PARP and active form of the caspase-3 in the induced radio-resistance rectal cancer cells may imply that the mechanism of radio-resistance is associated with resistance to cellular hypoxia. This notion was re-encapsulated by gene ontology analysis from the microarray. Most of the differently expressed genes in the radio-resistant rectal cancer cells were related to hypoxia, oxygen levels, oxidation reduction, regulation of epithelial cell differentiation, and cell-cell adhesion according to the gene ontology analysis (Table 1). The mechanistic comparison of resistance to radiation and hypoxia should be further investigated.

*ERRFI1* acts as a negative feedback regulator of the *EGF* receptor and is also known as *Mig6*, *RALT,* or *GENE33* [18]. *ERRFI1* directly interacts with four members of the *ERBB* family and works as a negative feedback regulator of the *ERBB* RTK pathway [19] by acting as a negative regulator of skin morphogenesis and tumor formation [9]. There has also been evidence that *ERRFI1* acts as a tumor-suppressor gene [20]. In various cancers such as skin, breast, pancreatic, and ovarian, *ERRFI1* has been strongly down-regulated. However, there have been no reports on this gene in colorectal cancer cell lines. In a recent study, it was discovered that the down-regulation of *ERRFI1* enhances resistance to *MEK* inhibition in *KRAS* mutant cancer cells [21]. In this present study, the basal mRNA and protein level of ERRFI1 was up-regulated in the induced radio-resistant human rectal cancer cell lines. Nevertheless, radio-resistant rectal cells showed increased proliferation rates after *ERRFI1* was down-regulated by siRNA (Fig. 5c, d). As mentioned previously, *ERRFI1* is known to work as a negative feedback regulator of the *ERBB* RTK pathway, so attenuated *ERRFI1* might increase the *ERBB* RTK pathway and lead to increased cell growth.

The N-myc downstream-regulated gene 1(NDRG1) has been reported as a potential metastasis suppressor by maintaining the localization of E-cadherin and β-catenin in prostate and colon cancer cells [13]. In addition, the neuroblastoma study revealed that NDRG1 is associated with increased level of resistant-related proteins such as MDR, LRP-1, and MRP-1 [14]. According to HPA dataset from The Human Protein Atlas, the protein expression of NDRG1 in normal rectum tissue was relatively low (24th out of 37 organs tested). On the other hands, the protein expression of NDRG1 in colorectal cancer was medium to high (8th out of 20 organs tested). Although few studies have shown that NDRG1 expression may be associated with less aggressive colorectal cancer [22, 23], association between NDRG1 and prognostic features in rectal cancer remained unknown [24]. Moreover, the relationship between the protein expression of NDRG1 and resistance to ionizing radiation in rectal cancer cell lines has not been studied.

In the present study, both mRNA (Fig. 3a) and protein expression (Fig. 6a) of NDRG1 were significantly augmented when SNU-283 and SNU-503 cells acquired resistance to ionizing radiation. Phosphorylation of gamma H2AX has been a biomarker for DNA double-strand breaks [25]. Immunocytochemistry revealed that both active forms of caspase-3 and phosphorylated gamma H2AX were decreased in SNU-503R80Gy cells compared to SNU-503 cells. When NDRG1 was down-regulated in SNU-503R80Gy, cells were damaged again from ionizing radiation and both active forms of caspase-3 and phosphorylated gamma H2AX were detected in a similar level with SNU-503, naïve cells (Figs. 7 and 8). Western Blot analysis revalidated that SNU-503R80Gy with down-regulated NDRG1 was more sensitive to initial damage induction (6–24 h after irradiation) than SNU-503R80Gy parental cells (Figs. 6c and 7). After 72 h, SNU-503R80Gy parental cells repaired damage more effectively than SNU-503R80Gy shNDRG1 cells (Figs. 6c and 8). This may imply that down-regulation of NDRG1 re-sensitized SNU-503R80Gy cells by disrupting rapid DNA damage recovery mechanism of radio-resistant rectal cancer cells. This was re-confirmed by time-dependent cell proliferation assay. Until 72 h after irradiation, the proliferation rate of SNU-503R80Gy cells with mock vector and shNDRG1 was similar. In 96 h, the proliferation rate of SNU-503R80Gy shNDRG1 was declined significantly (Fig. 6d).

Radio-resistance has been known to be associated with DNA damage repair mechanism. Glioma study revealed that DNA damage repair activity of Ape1/Ref-1 determined radiation resistance in glioma cells as there was a dose-dependent relationship between increasing Ape1 overexpression and increasing radio-resistance [26]. The role of NDRG1 in response to DNA damage has not reached an agreement. Siavash K. Kurdistani reported that DNA-damaging agents induced NDRG1 expression in a p53-dependent manner but independent of a p53-mediated G1 arrest. Beside, NDRG1 was re-localized from cytoplasmic region to the nucleus upon DNA damage [27]. On the other hands, other studies have shown no correlation between Ndrg-1 expression and DNA damage, despite the upregulation of p53 [28, 29].

Our study indicated that NDRG1-overexpressing SNU-503R80Gy cells began to recover from DNA damage after 72 h of irradiation. When the NDRG1 expression was down-regulated, the radio-resistant cells failed to recover from the DNA damage and the cell proliferation rate was decreased. We suggest that the augmented protein expression of NDRG1 is involved in DNA damage repair mechanism. Which DNA repair regulators are associated with NDRG1 should be further investigated.

## Conclusion

Taken together, we suggest that the higher NDRG1 expression may be associated with the radio-resistance of human rectal cancer cells, and it can be a potential therapeutic target for re-sensitizing radio-resistant rectal cancer cells to ionizing radiation.

## Additional file

Additional file 1: **Table S1.** Primers that were used for RT-PCR. **Table S2.** STR Profile of radio-resistant rectal cancer cell lines and their parental cell lines. (DOCX 27 kb)

## Abbreviations

ERRFI1: ERBB receptor feedback inhibitor 1
H19: Imprinted maternally expressed transcript (non-protein coding)
LRP-1: Low density lipoprotein receptor-related protein 1
MDR: Multidrug resistance gene
MPZL3: Myelin protein zero like 3
MRP-1: Multidrug resistance-associated protein 1
MTT: 3-(4,5-Dimethylthiazol-2-yl)-2,5-diphenyltetrazolium bromide
NDRG1: N-Myc downstream regulated 1
PARP: Poly ADP-ribose polymerase
TCF4: Transcription factor 4
UCA1: Urothelial cancer associated 1 (non-protein coding)
