# Supplementary material for: Identification of genes inducing resistance to ionizing radiation in human rectal cancer cell lines: re-sensitization of radio-resistant rectal cancer cells through down regulating NDRG1
Source: BMC Cancer. 2018 May 25;18:594. doi: 10.1186/s12885-018-4514-3 (PMC5970486; doi:10.1186/s12885-018-4514-3)
Supplement: Supplementary file 1 — Table S1. Primers that were used for RT-PCR. Table S2. STR Profile of radio-resistant rectal cancer cell lines and their parental cell lines. (DOCX 27 kb) [file 12885_2018_4514_MOESM1_ESM.docx]

**Table S1**. Primers that were used for RT-PCR

| Name of primers | Sequences |
| --- | --- |
| ERRFI1-F | 5'-GGC ACA ATG TCA ATA GCA GG-3' |
| ERRFI1-R | 5'-ATC GGA GCA GAT TTG GAA GC-3' |
| H19-F | 5'-TTG GCA GAC AGT ACA GCA TC-3' |
| H19-R | 5'-AGC CTA AGG TGT TCA GGA AG-3' |
| MPZL3-F | 5'-AGT CTT TCC AGT ACC CAA CC-3' |
| MPZL3-R | 5'-AGG GCA CAA AGA CAA GGA TG-3' |
| UCA1-F | 5'-AGC CTG TTT AGG TGG TCT TC-3' |
| UCA1-R | 5'-ACC GTA AGA GTT ACC CGA AG-3' |
| NDRG1-F | 5'-CCT GCA AGA GTT TGA TGT CC-3' |
| NDRG1-R | 5'-ACT CCA GGA AGC ATT TCA GC-3' |

**Table S2**. STR Profile of radio-resistant rectal cancer cell lines and their parental cell lines.

| **Cell-Name/**  **Loci** | **SNU-61** | **SNU-61R80Gy** | **SNU-283** | **SNU-283R80Gy** | **SNU-503** | **SNU-503R80Gy** |
| --- | --- | --- | --- | --- | --- | --- |
| D8S1179 | 14 | 14 | 14,15 | 14,15 | 10 | 10 |
| D21S11 | 29 | 29,32.2 | 29,30 | 29 | 30 | 30 |
| D7S820 | 8 | 8 | 11 | 11 | 10,11 | 10,11 |
| CSF1PO | 12 | 12 | 11,13 | 11,13 | 11 | 11 |
| D3S1358 | 15 | 15 | 15,18 | 15,18 | 15 | 15 |
| TH01 | 9 | 9 | 7,9 | 7,9 | 9 | 9 |
| D13S317 | 8 | 8 | 8,11 | 8,11 | 10 | 10 |
| D16S539 | 12 | 12 | 9,12 | 9,12 | 11,12 | 11,12 |
| D2S1338 | 18,19 | 18,19 | 17,18 | 17,18 | 23 | 23 |
| D19S433 | 12 | 12 | 14 | 14 | 13,14 | 13,14 |
| Vwa | 16 | 16 | 14,19 | 14,19 | 14,17 | 14,17 |
| TPOX | 8,9 | 8,9 | 8,11 | 8,11 | 8,11 | 8,11 |
| D18S51 | 16 | 16 | 15 | 15 | 15,16 | 15,16 |
| Amelogenin | X | X | X | X | X | X |
| D5S818 | 11 | 11 | 9 | 9 | 10 | 10 |
| FGA | 22,26 | 22,26 | 23,25 | 23 | 22 | 22 |
